# A nationwide study of metabolic syndrome prevalence in Iran; a comparative analysis of six definitions

**DOI:** 10.1371/journal.pone.0241926

**Published:** 2021-03-03

**Authors:** Ozra Tabatabaei-Malazy, Sahar Saeedi Moghaddam, Nazila Rezaei, Ali Sheidaei, Mohammad Javad Hajipour, Negar Mahmoudi, Zohreh Mahmoudi, Arezou Dilmaghani-Marand, Kamyar Rezaee, Mahdi Sabooni, Farideh Razi, Farzad Kompani, Alireza Delavari, Bagher Larijani, Farshad Farzadfar

**Affiliations:** 1 Non-Communicable Diseases Research Center, Endocrinology and Metabolism Population Sciences Institute, Tehran University of Medical Sciences, Tehran, Iran; 2 Endocrinology and Metabolism Research Center, Endocrinology and Metabolism Clinical Sciences Institute, Tehran University of Medical Sciences, Tehran, Iran; 3 Department of Epidemiology and Biostatistics, School of Public Health, Tehran University of Medical Sciences, Tehran, Iran; 4 Persian Gulf Marine Biotechnology Research Center, The Persian Gulf Biomedical Sciences Research Institute, Bushehr University of Medical Sciences, Bushehr, Iran; 5 Reference Health Laboratory, Ministry of Health and Medical Education, Tehran, Iran; 6 Diabetes Research Center, Endocrinology and Metabolism Clinical Sciences Institute, Tehran University of Medical Sciences, Tehran, Iran; 7 Division of Hematology and Oncology, Children’s Medical Center, Pediatrics Center of Excellence, Tehran University of Medical Sciences, Tehran, Iran; 8 Digestive Oncology Research Center, Digestive Disease Research Institute, Shariati Hospital, Tehran University of Medical Sciences, Tehran, Iran; Jiangsu Province Hospital of Chinese Medicine, CHINA

## Abstract

**Introduction:**

To integrate and execute a proper preventive plan and reduce the risk of non-communicable diseases (NCDs), policy makers need to have access to both reliable data and a unique definition of metabolic syndrome (MetS). This study was conducted on the data collected by cross-sectional studies of WHO’s STEPwise approach to surveillance of NCD risk factors (STEPs) to estimate the national and sub-national prevalence rates of MetS in Iran in 2016.

**Materials and methods:**

The prevalence of MetS was estimated among 18,414 individuals aged ≥25 years living in urban and rural areas of Iran using various definition criteria; National Cholesterol Education Program Adult Treatment Panel III 2004 (ATP III), International Diabetes Federation (IDF), American Heart Association/National Heart, Lung, and Blood Institute (AHA/NHLBI), Joint Interim Statement (JIS). Regional IDF (RIDF) and JIS (RJIS) were defined using ethnicity-specific values of waist circumference for the country.

**Results:**

National prevalence rate of MetS based on ATP III, IDF, AHA/NHLBI, JIS, RIDF and RJIS criteria were 38.3% (95% CI 37.4–39.1), 43.5% (42.7–44.4), 40.9% (40.1–41.8), 47.6% (46.8–48.5), 32.0% (31.2–32.9), and 40.8% (40.0–41.7), respectively. The prevalence was higher among females, in urban residents, and those aged 65–69 years. MetS was expected to affect about 18.7, 21.3, 20.0, 23.3, 15.7, and 20.0 million Iranians, respectively, based on ATP III, IDF, AHA/NHLBI, JIS, RIDF and RJIS. The two most common components noted in this population were reduced high-density lipoprotein cholesterol (HDL-C) levels and central obesity.

**Conclusion:**

High prevalence rate of MetS among Iranian adults is alarming, especially among females, urban residents, and the elderly. The JIS definition criteria is more appropriate to determine higher number of Iranians at risk of NCDs. Proper management and prevention of MetS is required to adopt multiple national plans including lifestyle modifications, medical interventions, and public education on NCDs risk factors.

## Introduction

The aging of the populations and global industrialization have resulted in an increase in the risk of non-communicable diseases (NCDs) such as cardiovascular disease (CVD) and type 2 diabetes mellitus (T2DM). Hence, the main goal of the World Health Organization’s (WHO) Global NCD Action Plan (25 by 25) and Sustainable Development Goal 4 (SDG4) is to reduce NCD-related mortality by 25% and 30% in 2025 and 2030, respectively [1,2]. Metabolic syndrome (MetS), which is a cluster of conditions including obesity, hypertension, hypertriglyceridemia, low level of high-density lipoprotein cholesterol (HDL-C), and glucose intolerance, has a positive association with the risk of CVD and T2DM [3].

Regardless of the definitions proposed for MetS [3], its prevalence is increasing in both developed and developing countries. The prevalence of MetS in various countries is affected by certain influential factors such as age, sex, central obesity, race, and urbanization [4]. A significant increase in the prevalence of MetS among US adults has been reported, changing from 29.2% in the third National Health and Nutrition Examination Survey (NHANES III) in 1988–1994 to 34.2% in the NHANES 1999–2006 [5]. However, its prevalence remained stable during 2007–2014 among US adults and the age-adjusted prevalence was reported at 34.3±0.8% (±standard error) [6].

The reported prevalence of MetS in Middle Eastern countries varies, ranging from 16.7% to 51% [7–10]. Despite the large body of growing evidence on its regional and global prevalence, there is a lack of reliable national data due to the scarcity of studies with good design and sufficient sample size as well as lack of unique definition criteria to compare its prevalence between countries [11]. The aim of this study was to estimate the national and sub-national prevalence rates of MetS and its components in Iran, as a country in Middle Eastern region, using STEPs 2016 data and introducing unique MetS definition criteria with coverage of greater part of affected Iranian populations to policymakers for reducing the risk of NCDs in a large sample size.

## Materials and methods

### Study design and participants

Methodology of the study is reported in details elsewhere [12]. As summary, using a systematic cluster random sampling frame, proportional to the adult population of each province, it was planned to collect data on 31,050 Iranian subjects (3,105 clusters) aged ≥18 years living in urban and rural areas of 31 provinces of Iran in 2016. In addition, 10% more than estimated sample size for each province was considered to control non-response error [12]. All Iranians aged ≥ 18 years who were living in Iran at the time of data collection were eligible for inclusion in the STEPs 2016 but taking blood samples were limited to individuals who were 25 years and above. Since most of variables/components for making MetS were taken from laboratory we excluded participants who aged less than 25 years old in current study. Data on subjects’ history of metabolic risk factors, physical measurements, and lab assessments were collected in three steps (Supporting information). Data on metabolic risk factors such as high fasting plasma glucose, hypertension and hyperlipidemia were collected using self-report or medical records (Step 1). Physical measurements included the assessment of anthropometric variables, blood pressure, and pedometer data (Step 2). Biochemical markers such as lipid profile, and glucose and glycated hemoglobin A1C (HbA1C) levels were measured on collected blood samples after overnight fasting (Step 3). All three of these sequential processes of data collection were completely computer-based and digitalized. The study was approved by the Ethics Committee of the National Institute for Medical Research Development (NIMAD) under the following ID number: IR.NIMAD.REC.1394.032.

### Data collection and measurements

In STEPs 2016, data were collected from Iranian residents who had completed a written informed consent. Past medical history, currently used medications, and physical examination results were also recorded. The Global Physical Activity Questionnaire (GPAQ) was used to calculate physical activity as the Metabolic Equivalents (MET) value in minutes per week for work, recreation, and transport domains. The WHO’s recommendation was considered to define low physical activity as less than 600 MET minutes per week [13]. Anthropometric indices including height, weight, Body Mass Index (BMI), waist circumference (WC), hip circumference (HC), and waist-to-hip-ratio (WHR) were measured as recommended by standard protocols proposed in the STEPs manual [14]. Height was measured using a tape (0.5 cm error) in standing position with bare foot. Weight was measured using innofit, JY-218A personal scale, SN: 14010936 (China). BMI was calculated through dividing weight (kg) by height in squared meters (m^2^). The broadest area between the edge of the lower ribs and the iliac crest was measured in order to obtain WC. Moreover, the HC was measured around the fullest part of the buttocks at a horizontal plane while the subjects were wearing nonrestrictive underwear and lightweight pants or skirts [14]. Physical measurements including Systolic Blood Pressure (SBP) and Diastolic Blood Pressure (DBP) were also measured based on the recommended protocol in the STEPs manual [14]. They were measured three times using Beurer medical, Type: BM 20, Art_Nr: 652.11 (Germany). The mean values of the second and third readings were used for analysis.

### Laboratory tests

Venous blood samples were collected after 12–14 hours of overnight fasting to measure serum levels of fasting blood sugar (FBS), total cholesterol (TChol), HDL-C, low density lipoprotein cholesterol (LDL-C), and triglycerides (TGs). The collected samples were stored under standard conditions (at temperatures lower than 4°C) in vaccine transfer boxes and transferred to the central processing/archiving laboratory of study in the Non-Communicable Diseases Research Center (NCDRC) of Tehran University of Medical Sciences in the shortest possible time (less than 18 hours).

During the transfer, a digital thermometer recorded the temperature in each cold box. FBS, TChol, and TGs levels were measured following a standard enzymatic method using an auto-analyzer (Cobas C31, Hitachi, Japan). In patients with TGs <400 mg/dl, LDL-C levels was estimated by the Friedewald formula (TChol minus HDL-C minus TGs/5 in mg/dl), whereas it was measured directly in the others [15]. HDL-C levels were measured using the homogeneous enzymatic cardiometric test. All the collected samples were assessed using a kit and definite batch numbers or devices in the NCDRC laboratory.

### Definitions

Table 1 presents details on the six criteria used to define MetS: the National Cholesterol Education Program Adult Treatment Panel III (ATP III), the International Diabetes Federation (IDF), the American Heart Association/National Heart, Lung, and Blood Institute (AHA/NHLBI), and the Joint Interim Statement (JIS) [3,16–18]. According to the recommendations of the Iranian National Committee of Obesity, the ethnicity-specific values were applied in the IDF and JIS definitions [19]. In this regard, the Iranian cutoffs for WC were introduced as Regional IDF (RIDF) and Regional JIS (RJIS), respectively.

**Table 1:** Diagnostic criteria used to define MetS.

Components	ATP III	IDF	Regional IDF	AHA/NHLBI	JIS	Regional JIS
**Glucose domain**	FBS≥100 mg/dl (Includes diabetes)	FBS≥100 mg/dl (Includes diabetes)	FBS≥100 mg/dl (Includes diabetes)	FBS≥100 mg/dl (Includes diabetes)	FBS≥100 mg/dl (Includes diabetes)	FBS≥100 mg/dl (Includes diabetes)
**Obesity domain**	WC≥102 cm men/≥88 cm (women)	WC≥94 cm men/≥80 cm (women)	WC≥95 cm (for Iranian men & women)	WC≥102 cm men/≥88 cm (women)	WC≥94 cm men/≥80 cm (women)	WC≥95 cm (for Iranian men & women)
**Lipid profile**	TGs≥150 mg/dl	TGs≥150 mg/dl or under treatment	TGs≥150 mg/dl or under treatment	TGs≥150 mg/dl or under treatment	TGs≥150 mg/dl or under treatment	TGs≥150 mg/dl or undertreatment
	HDL-C<40 mg/dl (men)/<50 mg/dl (women)	HDL-C<40 mg/dl (men)/<50 mg/dl (women) or under treatment	HDL-C<40 mg/dl (men)/<50 mg/dl (women) or under treatment	HDL-C<40 mg/dl (men)/<50 mg/dl (women) or under treatment	HDL-C<40 mg/dl (men)/<50 mg/dl (women) or under treatment	HDL-C<40 mg/dl (men)/<50 mg/dl (women) or undertreatment
**BP domain**	SBP≥130 mmHg or DBP≥85 mmHg	SBP≥130 mmHg or DBP≥85 mmHg or under treatment	SBP≥130 mmHg or DBP≥85 mmHg or under treatment	SBP≥130 mmHg or DBP≥85 mmHg or under treatment	SBP≥130 mmHg or DBP≥85 mmHg or under treatment	SBP≥130 mmHg or DBP≥85 mmHg or undertreatment
**MetS definition**	Any 3 of the above components	Obesity domain plus any 2 of the above components	Obesity domain plus any 2 of the above components	Any 3 of the above components	Any 3 of the above components	Any 3 of the above components

**ATP III:** National Cholesterol Education Program Adult Treatment Panel III; **IDF:** International Diabetes Federation; **AHA/NHLBI:** American Heart Association/National Heart, Lung, and Blood Institute; **JIS:** Joint Interim Statement; **FBS:** Fasting Blood Sugar; **WC:** Waist circumference; **TGs:** Triglycerides; **HDL-C:** High Density Lipoprotein Cholesterol; **SBP:** Systolic Blood Pressure; **DBP:** Diastolic Blood Pressure; **MetS:** Metabolic Syndrome.

### Data analysis

In this study, the data of 18,414 individuals aged ≥25 years old who participated in all the three steps and had reliable results based on the STEPs protocol and no missings in the items relevant to the MetS defintitions were used [14]. Applying complex survey weights, as described previously [12], the estimated prevalence rates standardized for sex, area of residence, and age subgroups (25–34, 35–44, 45–54, 55–64, 65–69, 70+) were reported for national and sub–national levels with 95% confidence intervals (95% CI). The burden of population was calculated as the number of people from each gender and residency area who have MetS based on each definition criteria.

The statistical analyses were performed using STATA version 12 and R statistical packages version 3.4.0. P-value ≤ 0.05 was set as statistically significant. Age-standardization of sub-national results was performed based on the National Population and Housing Census conducted by the Statistical Center of Iran in 2016 [20]. The level of agreement between different definitions of MetS, ranging from −1 to +1, was calculated through the Cohen’s Kappa statistic. A value of 1 indicated perfect agreement, while a value of 0 represented that the agreement was the result of no better than chance [21].

## Results

The study was conducted on 18,414 participants including 8,570 males and 9,844 females aged between 25 and 70+ years (mean age: 47.4, 95% CI: 47.2–47.6). Table 2 presents the overall and sex-based demographic and biochemical characteristics of the included subjects.

**Table 2:** Socio-demographic, lifestyle and clinical characteristics of the participants.

Variable	Total (95% CI)	Males (95% CI)	Females (95% CI)	P-value
**Mean Age (yr)**	47.4 (47.2–47.6)	48.0 (47.6–48.3)	47.0 (46.7–47.3)	<0.001
**Years of education (%)**				
Illiterate	18.2 (17.6–18.8)	12.3 (11.6–13.0)	23.4 (22.5–24.2)	<0.001
Primary	29.1 (28.4–29.8)	28.1 (27.1–29.0)	30.0 (29.1–31.0)	<0.001
7–12 years	36.7 (35.9–37.4)	41.5 (40.4–42.6)	32.4 (31.5–33.4)	0.004
Academic	16.0 (15.5–16.6)	18.1 (17.3–19.0)	14.2 (13.5–14.9)	<0.001
**Job (%)**				
Employed	38.8 (38.1–39.6)	73.4 (72.4–74.3)	8.7 (8.1–9.3)	<0.001
Housewife	46.1 (45.4–46.9)	1.0 (0.8–1.2)	85.5 (84.8–86.2)	<0.001
Unemployed	15.0 (14.5–15.6)	25.6 (24.7–26.6)	5.8 (5.3–6.3)	<0.001
**Marital Status (%)**				
Divorced	1.8 (1.6–2.0)	0.7 (0.5–0.9)	2.8 (2.5–3.1)	<0.001
Married	83.2 (82.6–83.8)	89.9 (89.2–90.5)	77.4 (76.5–78.2)	<0.001
Never married	8.0 (7.5–8.4)	8.2 (7.6–8.8)	7.7 (7.2–8.3)	0.278
Widowed	7.0 (6.6–7.4)	1.2 (1.0–1.5)	12.1 (11.4–12.7)	<0.001
**Self-reported medication for (%)**				
Diabetes	13.5 (12.7–14.2)	10.0 (9.1–10.9)	17.5 (16.3–18.7)	<0.001
Hypertension	67.6 (66.0–69.2)	64.4 (61.7–67.0)	69.6 (67.6–71.7)	0.002
Dyslipidemia	31.3 (30.6–32.0)	33.7 (32.7–34.8)	29.2 (28.3–30.2)	<0.001
**Low Physical activity (<600 METs) (%)**	55.7 (54.9–56.5)	47.2 (46.0–48.4)	62.0 (61.0–63.0)	<0.001
**WC (cm)**	92.2 (92.0–92.4)	92.2 (91.9–92.5)	92.1 (91.8–92.4)	0.552
WHR (*100)	90.7 (90.5–90.8)	93.0 (92.8–93.2)	88.6 (88.4–88.8)	<0.001
**Weight (kg)**	71.9 (71.7–72.2)	75.4 (75.1–75.7)	68.9 (68.6–69.2)	<0.001
**BMI (kg/m**^**2**^**)**	27.0 (26.9–27.1)	25.9 (25.8–26.0)	28.0 (27.9–28.1)	<0.001
**SBP (mmHg)**	126.9 (126.7–127.2)	127.1 (126.7–127.5)	126.8 (126.3–127.2)	0.200
**DBP (mmHg)**	77.8 (77.6–77.9)	78.4 (78.2–78.6)	77.2 (77.0–77.5)	<0.001
**FBS (mg/dl)**	99.1 (98.5–99.8)	98.7 (97.7–99.6)	99.5 (98.7–100.4)	0.194
**TChol (mg/dl)**	164.6 (164.0–165.2)	161.1 (160.2–162.0)	167.5 (166.7–168.3)	<0.001
**TGs (mg/dl)**	129.0 (127.6–130.4)	136.8 (134.7–138.8)	122.4 (120.5–124.3)	<0.001
**LDL-C (mg/dl)**	97.7 (97.2–98.3)	96.1 (95.3–96.8)	99.2 (98.4–99.9)	<0.001
**HDL-C (mg/dl)**	41.2 (41.0–41.4)	37.9 (37.6–38.1)	44.0 (43.7–44.2)	<0.001

**WC:** Waist circumference; **WHR**: Waist Hip Ratio; **BMI:** Body Mass Index; **SBP:** Systolic Blood Pressure; **DBP:** Diastolic Blood Pressure; **FBS:** Fasting Blood Sugar; **TChol:** Total Cholesterol; **TGs:** Triglycerides; **LDL-C:** Low Density Lipoprotein Cholesterol; **HDL-C:** High Density Lipoprotein Cholesterol.

-Data are presented as point estimates and 95% CI.

-P≤ 0.05 was considered as statistically significant by survey logistic or linear regression models.

**Fig 1:**
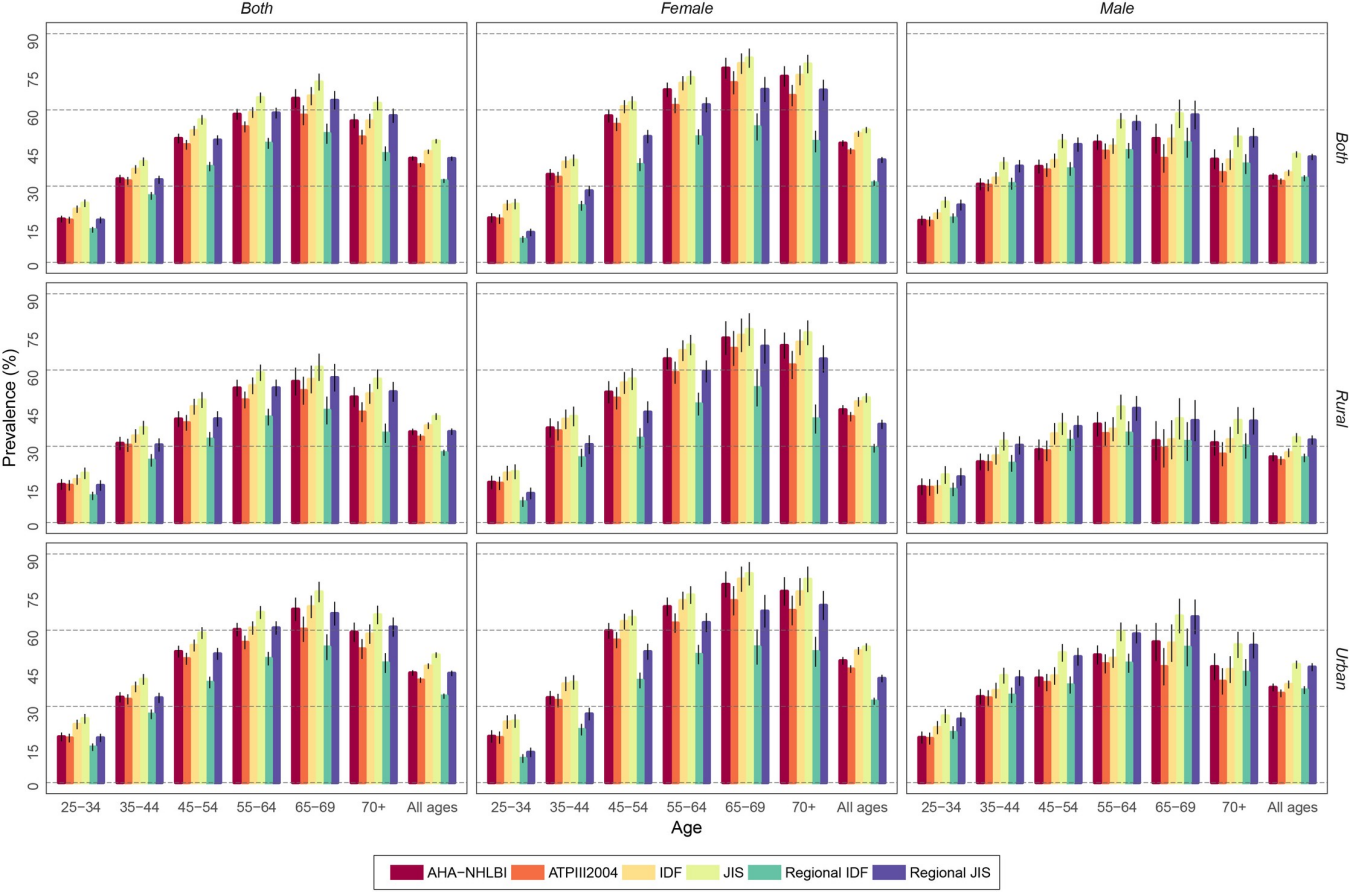
Age trends of MetS among the Iranian population based on sex and area of residence.

The estimated overall prevalence of MetS for all age groups, irrespective of sex or area of residence, was 32.0–47.6% using various criteria. The highest rate (47.6%) was estimated based on JIS (Table 3). The estimated national burden of MetS for all ages was 7.6–10.5 and 7.1–12.7 million persons among males and females, respectively. The rate was about 11.7–18.4 and 3.0–4.9 million persons in urban and rural residents, correspondingly (Table 3). The national prevalence rate was higher among older age groups, with the highest spike reported in the 65–69 age group regardless of the definition criteria (Fig 1).

**Table 3:** National prevalence and burden of MetS in Iranian adults aged ≥25 years based on various MetS definitions (for all ages).

Sex	Area	ATP III		IDF		AHA/NHLBI		JIS		Regional IDF		Regional JIS	
		Prevalence (%, 95% CI)	Burden* (%, 95% CI)	Prevalence (%, 95% CI)	Burden* (%, 95% CI)	Prevalence (%, 95% CI)	Burden* (%, 95% CI)	Prevalence (%, 95% CI)	Burden* (%, 95% CI)	Prevalence (%, 95% CI)	Burden* (%, 95% CI)	Prevalence (%, 95% CI)	Burden* (%, 95% CI)
**Both**	**Both**	38.3 (37.4–39.1)	18.7 (18.3–19.1)	43.5 (42.7–44.4)	21.3 (20.9–21.7)	40.9 (40.1–41.8)	20.0 (19.6–20.4)	47.6 (46.8–48.5)	23.3 (22.9–23.7)	32.0 (31.2–32.9)	15.7 (15.3–16.1)	40.8 (40.0–41.7)	20.0 (19.5–20.4)
	**Rural**	33.5 (32.2–34.8)	3.9 (3.8–4.0)	38.1 (36.8–39.4)	4.5 (4.3–4.6)	35.6 (34.3–36.9)	4.2 (4.0–4.3)	41.7 (40.3–43.0)	4.9 (4.7–5.0)	27.4 (26.2–28.6)	3.2 (3.1–3.3)	35.7 (34.4–37.0)	4.2 (4.0–4.3)
	**Urban**	40.3 (39.3–41.4)	14.8 (14.5–15.1)	45.8 (44.7–46.9)	16.8 (16.5–17.1)	43.1 (42.1–44.2)	15.8 (15.6–16.1)	50.2 (49.1–51.3)	18.4 (18.1–18.7)	34.0 (33.0–35.0)	12.5 (12.2–12.7)	43.0 (41.9–44.1)	15.8 (15.5–16.1)
**Female**	**Both**	43.8 (42.6–45.0)	10.8 (10.6–11.0)	50.6 (49.4–51.8)	12.5 (12.3–12.7)	46.9 (45.7–48.1)	11.5 (11.3–11.7)	52.1 (50.9–53.3)	12.7 (12.5–12.9)	31.2 (30.1–32.3)	7.5 (7.4–7.7)	40.3^†^ (39.1–41.4)	9.8 (9.6–10.0)
	**Rural**	41.6 (39.8–43.5)	2.5 (2.4–2.5)	47.5 (45.6–49.3)	2.8 (2.7–2.9)	44.3 (42.5–46.2)	2.6 (2.6–2.7)	49.0 (47.2–50.9)	2.9 (2.8–2.9)	29.2 (27.5–30.9)	1.7 (1.6–1.8)	38.6 (36.8–40.4)	2.2 (2.2–2.3)
	**Urban**	44.7 (43.2–46.2)	8.3 (8.2–8.4)	51.9 (50.4–53.4)	9.7 (9.5–9.8)	47.9 (46.4–49.4)	8.9 (8.8–9.0)	53.3 (51.8–54.8)	9.9 (9.7–10.0)	32.0 (30.6–33.4)	5.8 (5.7–6.0)	41.0 (39.5–42.4)	7.5 (7.4–7.6)
**Male**	**Both**	31.8 (30.6–33.0)	7.9 (7.7–8.2)	35.2 (34.0–36.4)	8.8 (8.6–9.0)	33.9 (32.7–35.1)	8.5 (8.2–8.7)	42.4 (41.2–43.7)	10.5 (10.3–10.8)	33.1 (31.9–34.3)	8.1 (7.9–8.3)	41.5^†^ (40.2–42.7)	10.2 (10.0–10.4)
	**Rural**	24.3 (22.6–26.0)	1.5 (1.4–1.5)	27.4 (25.7–29.1)	1.6 (1.6–1.7)	25.8 (24.1–27.5)	1.5 (1.5–1.6)	33.3 (31.5–35.2)	2.0 (1.9–2.1)	25.4 (23.7–27.1)	1.5 (1.4–1.6)	32.4 (30.6–34.2)	1.9 (1.8–2.0)
	**Urban**	35.1 (33.6–36.6)	6.5 (6.3–6.6)	38.6 (37.0–40.2)	7.2 (7.0–7.3)	37.4 (35.9–39.0)	6.9 (6.8–7.1)	46.4 (44.8–48.0)	8.6 (8.4–8.7)	36.4 (34.9–38.0)	6.6 (6.5–6.8)	45.4 (43.8–47.0)	8.3 (8.1–8.4)

**ATP III:** National Cholesterol Education Program Adult Treatment Panel III; **IDF:** International Diabetes Federation; **AHA/NHLBI:** American Heart Association/National Heart, Lung, and Blood Institute; **JIS:** Joint Interim Statement; **MetS:** Metabolic Syndrome; **95% CI:** 95% Confidence Interval.

-Data are presented as point estimates and 95% CI.

*Rounded to the nearest million.

^†^P value was statistically non-significant (P = 0.173) using Chi-Square analysis, but other P-values were statistically significant (P≤0.05).

The highest prevalence rate of MetS were estimated in uneducated subjects (61.5%) and those with low physical activity (51.8%) (Table 4). Based on all criteria, the most common components of MetS reduced in this population were low HDL-C levels (<40 mg/dl in males and <50 mg/dl in females) and the obesity domain, at 69.3–71.1%, and 44.1–66.9%, respectively (Table 5).

**Table 4:** National prevalence of MetS among Iranian adults aged ≥25 years based on physical activity and education levels (for all ages).

Variable	Participants	Category	ATP III		IDF		AHA/NHLBI		JIS		Regional IDF		Regional JIS	
			Prevalence	P-value	Prevalence	P-value	Prevalence	P-value	Prevalence	P-value	Prevalence	P-value	Prevalence	P-value
**Low physical activity (Less than 600 METs)**	**Total**	**Yes**	42.3 (41.1–43.6)	<0.001	48.2 (47.0–49.4)	<0.001	45.4 (44.1–46.6)	<0.001	51.8 (50.6–53.1)	<0.001	34.9 (33.7–36.1)	<0.001	43.9 (42.7–45.1)	<0.001
		**No**	36.3 (35.0–37.7)		41.2 (39.9–42.6)		38.7 (37.4–40.1)		45.7 (44.4–47.1)		30.7 (29.4–32.0)		39.5 (38.1–40.8)	
	**Rural**	**Yes**	39.7 (37.6–41.8)	<0.001	45.5 (43.3–47.6)	<0.001	42.1 (40.0–44.3)	<0.001	48.4 (46.3–50.6)	<0.001	31.6 (29.6–33.6)	0.001	40.8 (38.7–42.9)	<0.001
		**No**	32.5 (30.6–34.4)		36.5 (34.6–38.4)		34.8 (32.9–36.7)		40.5 (38.5–42.4)		26.9 (25.1–28.7)		34.9 (33.0–36.8)	
	**Urban**	**Yes**	43.2 (41.7–44.6)	<0.001	49.1 (47.6–50.5)	<0.001	46.4 (44.9–47.9)	<0.001	52.9 (51.4–54.4)	<0.001	35.9 (34.5–37.4)	0.003	44.9 (43.4–46.4)	0.005
		**No**	38.2 (36.4–39.9)		43.5 (41.7–45.3)		40.6 (38.9–42.4)		48.2 (46.4–50.0)		32.5 (30.8–34.2)		41.6 (39.9–43.4)	
	**Female**	**Yes**	44.9 (43.4–46.5)	0.040	52.1 (50.5–53.6)	0.004	48.2 (46.7–49.8)	0.015	53.5 (51.9–55.0)	0.008	32.4 (31.0–33.9)	0.018	41.4 (39.9–43.0)	0.031
		**No**	42.3 (40.4–44.2)		48.4 (46.5–50.4)		45.1 (43.1–47.0)		50.0 (48.1–52.0)		29.6 (27.8–31.4)		38.7 (36.8–40.6)	
	**Male**	**Yes**	37.7 (35.7–39.7)	<0.001	41.2 (39.2–43.2)	<0.001	40.3 (38.2–42.3)	<0.001	48.9 (46.9–50.9)	<0.001	39.4 (37.4–41.3)	<0.001	48.4 (46.4–50.4)	<0.001
		**No**	30.5 (28.7–32.2)		34.1 (32.3–36.0)		32.5 (30.7–34.3)		41.5 (39.6–43.4)		31.7 (29.9–33.6)		40.2 (38.3–42.1)	
**Level of education**	**Total**	**Illiterate**	51.0 (49.1–52.9)		57.4 (55.5–59.2)		55.7 (53.8–57.5)		61.5 (59.7–63.3)		42.0 (40.2–43.9)		54.3 (52.4–56.1)	
		**Primary**	42.5 (41.0–44.1)	<0.001	48.0 (46.4–49.6)	<0.001	45.7 (44.1–47.3)	<0.001	51.9 (50.3–53.4)	<0.001	36.3 (34.7–37.9)	<0.001	45.1 (43.5–46.7)	<0.001
		**7–12 years**	33.2 (31.8–34.6)	<0.001	37.7 (36.3–39.2)	<0.001	34.8 (33.4–36.2)	<0.001	42.0 (40.6–43.5)	<0.001	27.0 (25.7–28.3)	<0.001	35.0 (33.6–36.4)	<0.001
		**Academic**	29.6 (27.6–31.6)	<0.001	34.7 (32.6–36.9)	<0.001	31.5 (29.4–33.6)	<0.001	38.9 (36.8–41.1)	<0.001	25.9 (24.0–27.9)	<0.001	32.8 (30.8–34.9)	<0.001
	**Rural**	**Illiterate**	43.9 (41.5–46.3)		50.2 (47.8–52.6)		48.0 (45.6–50.3)		54.0 (51.7–56.4)		35.4 (33.1–37.8)		47.3 (44.9–49.7)	
		**Primary**	32.1 (29.9–34.3)	<0.001	36.3 (34.1–38.5)	<0.001	34.1 (31.9–36.3)	<0.001	39.8 (37.5–42.0)	<0.001	26.9 (24.9–28.9)	<0.001	34.0 (31.8–36.2)	<0.001
		**7–12 years**	26.3 (23.9–28.7)	<0.001	29.4 (26.9–31.8)	<0.001	26.9 (24.5–29.3)	<0.001	33.3 (30.8–35.8)	<0.001	21.2 (19.0–23.4)	<0.001	27.6 (25.2–30.0)	<0.001
		**Academic**	25.8 (21.8–29.9)	<0.001	30.5 (26.3–34.8)	<0.001	26.8 (22.7–30.9)	<0.001	32.9 (28.5–37.2)	<0.001	21.5 (17.8–25.3)	<0.001	28.2 (24.1–32.4)	<0.001
	**Urban**	**Illiterate**	58.6 (55.8–61.5)		65.1 (62.3–67.8)		64.0 (61.2–66.8)		69.6 (66.9–72.2)		49.1 (46.2–52.0)		61.8 (59.0–64.6)	
		**Primary**	48.5 (46.4–50.7)	<0.001	54.7 (52.5–56.8)	<0.001	52.3 (50.2–54.4)	<0.001	58.8 (56.7–60.9)	<0.001	41.7 (39.5–43.8)	<0.001	51.4 (49.3–53.5)	<0.001
		**7–12 years**	35.0 (33.4–36.7)	<0.001	40.0 (38.3–41.7)	<0.001	36.9 (35.3–38.6)	<0.001	44.4 (42.7–46.1)	<0.001	28.6 (27.1–30.2)	<0.001	37.1 (35.4–38.7)	<0.001
		**Academic**	30.3 (28.0–32.5)	<0.001	35.5 (33.1–37.9)	<0.001	32.3 (30.0–34.7)	<0.001	40.0 (37.6–42.4)	<0.001	26.7 (24.5–28.9)	<0.001	33.7 (31.4–36.0)	<0.001
	**Female**	**Illiterate**	60.3 (58.1–62.5)		67.2 (65.1–69.3)		65.5 (63.4–67.7)		69.8 (67.7–71.8)		45.6 (43.3–47.9)		59.6 (57.4–61.8)	
		**Primary**	49.7 (47.5–51.9)	<0.001	56.6 (54.4–58.7)	<0.001	53.3 (51.2–55.5)	<0.001	57.9 (55.8–60.0)	<0.001	37.5 (35.3–39.6)	<0.001	46.4 (44.2–48.6)	<0.001
		**7–12 years**	35.0 (32.9–37.1)	<0.001	42.2 (40.1–44.4)	<0.001	36.9 (34.8–39.1)	<0.001	43.1 (41.0–45.3)	<0.001	22.6 (20.7–24.4)	<0.001	29.9 (27.9–31.9)	<0.001
		**Academic**	27.7 (24.8–30.5)	<0.001	33.2 (30.2–36.2)	<0.001	29.2 (26.3–32.1)	<0.001	34.7 (31.7–37.8)	<0.001	17.2 (14.8–19.5)	<0.001	23.4 (20.8–26.0)	<0.001
	**Male**	**Illiterate**	30.6 (27.4–33.7)		35.8 (32.6–39.0)		34.0 (30.8–37.2)		43.3 (40.0–46.6)		34.1 (30.9–37.4)		42.5 (39.2–45.8)	
		**Primary**	33.4 (31.1–35.6)	0.157	37.0 (34.7–39.3)	0.539	35.9 (33.6–38.2)	0.348	44.1 (41.8–46.5)	0.690	34.8 (32.5–37.1)	0.735	43.3 (41.0–45.7)	0.700
		**7–12 years**	31.4 (29.6–33.2)	0.650	33.4 (31.6–35.3)	0.216	32.7 (30.8–34.5)	0.490	41.0 (39.1–42.9)	0.230	31.3 (29.5–33.1)	0.130	40.0 (38.1–41.9)	0.197
		**Academic**	31.3 (28.5–34.1)	0.720	36.1 (33.1–39.1)	0.889	33.5 (30.7–36.4)	0.839	42.6 (39.6–45.6)	0.762	33.7 (30.8–36.6)	0.850	41.3 (38.3–44.3)	0.591

- Prevalence rates are presented as point estimates (%) and 95% CI.

- Low physical activity and literacy level were considered as references.

- P≤ 0.05 was considered as statistically significant by survey linear regression analysis.

**Table 5:** National prevalence of MetS’ components in Iranian adults aged ≥25 years based on various definitions (for all ages).

Components	Sex	Area	ATP III (%, 95% CI)	IDF (%, 95% CI)	AHA/NHLBI (%, 95% CI)	JIS (%, 95% CI)	Regional IDF (%, 95% CI)	Regional JIS (%, 95% CI)
**Glucose domain**	**Both**	**Both**	29.1 (28.3–29.9)	29.1 (28.3–29.9)	29.1 (28.3–29.9)	29.1 (28.3–29.9)	29.1 (28.3–29.9)	29.1 (28.3–29.9)
		**Rural**	25.2 (24.0–26.4)	25.2 (24.0–26.4)	25.2 (24.0–26.4)	25.2 (24.0–26.4)	25.2 (24.0–26.4)	25.2 (24.0–26.4)
		**Urban**	30.8 (29.8–31.8)	30.8 (29.8–31.8)	30.8 (29.8–31.8)	30.8 (29.8–31.8)	30.8 (29.8–31.8)	30.8 (29.8–31.8)
	**Female**	**Both**	29.1^†^ (28.0–30.2)	29.1^†^ (28.0–30.2)	29.1^†^ (28.0–30.2)	29.1^†^ (28.0–30.2)	29.1^†^ (28.0–30.2)	29.1^†^ (28.0–30.2)
		**Rural**	27.5 (25.8–29.1)	27.5 (25.8–29.1)	27.5 (25.8–29.1)	27.5 (25.8–29.1)	27.5 (25.8–29.1)	27.5 (25.8–29.1)
		**Urban**	29.8 (28.4–31.2)	29.8 (28.4–31.2)	29.8 (28.4–31.2)	29.8 (28.4–31.2)	29.8 (28.4–31.2)	29.8 (28.4–31.2)
	**Male**	**Both**	29.2^†^ (28.0–30.3)	29.2^†^ (28.0–30.3)	29.2^†^ (28.0–30.3)	29.2^†^ (28.0–30.3)	29.2^†^ (28.0–30.3)	29.2^†^ (28.0–30.3)
		**Rural**	22.6 (21.0–24.3)	22.6 (21.0–24.3)	22.6 (21.0–24.3)	22.6 (21.0–24.3)	22.6 (21.0–24.3)	22.6 (21.0–24.3)
		**Urban**	32.0 (30.5–33.5)	32.0 (30.5–33.5)	32.0 (30.5–33.5)	32.0 (30.5–33.5)	32.0 (30.5–33.5)	32.0 (30.5–33.5)
**Obesity domain**	**Both**	**Both**	44.5 (43.8–45.3)	66.9 (66.2–67.6)	44.5 (43.8–45.3)	66.9 (66.2–67.6)	44.1 (43.3–44.8)	44.1 (43.3–44.8)
		**Rural**	40.3 (39.0–41.5)	60.2 (59.0–61.5)	40.3 (39.0–41.5)	60.2 (59.0–61.5)	38.6 (37.4–39.9)	38.6 (37.4–39.9)
		**Urban**	46.8 (45.9–47.7)	70.4 (69.6–71.3)	46.8 (45.9–47.7)	70.4 (69.6–71.3)	47.0 (46.0–47.9)	47.0 (46.0–47.9)
	**Female**	**Both**	63.7 (62.7–64.7)	83.0 (82.2–83.8)	63.7 (62.7–64.7)	83.0 (82.2–83.8)	43.1 (42.1–44.2)	43.1 (42.1–44.2)
		**Rural**	60.9 (59.2–62.6)	79.5 (78.1–80.9)	60.9 (59.2–62.6)	79.5 (78.1–80.9)	41.1 (39.4–42.8)	41.1 (39.4–42.8)
		**Urban**	65.1 (63.9–66.3)	84.8 (83.9–85.7)	65.1 (63.9–66.3)	84.8 (83.9–85.7)	44.2 (42.9–45.4)	44.2 (42.9–45.4)
	**Male**	**Both**	22.7 (21.7–23.6)	48.5 (47.4–49.6)	22.7 (21.7–23.6)	48.5 (47.4–49.6)	45.2 (44.1–46.2)	45.2 (44.1–46.2)
		**Rural**	17.5 (16.0–19.0)	39.0 (37.2–40.8)	17.5 (16.0–19.0)	39.0 (37.2–40.8)	35.9 (34.1–37.7)	35.9 (34.1–37.7)
		**Urban**	25.5 (24.3–26.7)	53.7 (52.3–55.1)	25.5 (24.3–26.7)	53.7 (52.3–55.1)	50.2 (48.8–51.6)	50.2 (48.8–51.6)
**TGs domain**	**Both**	**Both**	27.7 (26.9–28.4)	32.0 (31.2–32.8)	32.0 (31.2–32.8)	32.0 (31.2–32.8)	32.0 (31.2–32.8)	32.0 (31.2–32.8)
		**Rural**	22.4 (21.3–23.5)	25.9 (24.7–27.0)	25.9 (24.7–27.0)	25.9 (24.7–27.0)	25.9 (24.7–27.0)	25.9 (24.7–27.0)
		**Urban**	29.9 (28.9–30.9)	34.6 (33.5–35.6)	34.6 (33.5–35.6)	34.6 (33.5–35.6)	34.6 (33.5–35.6)	34.6 (33.5–35.6)
	**Female**	**Both**	24.5 (23.5–25.5)	30.0 (28.9–31.1)	30.0 (28.9–31.1)	30.0 (28.9–31.1)	30.0 (28.9–31.1)	30.0 (28.9–31.1)
		**Rural**	20.4 (18.9–22.0)	25.0^††^ (23.4–26.6)	25.0^††^ (23.4–26.6)	25.0^††^ (23.4–26.6)	25.0^††^ (23.4–26.6)	25.0^††^ (23.4–26.6)
		**Urban**	26.2 (24.8–27.5)	32.0 (30.6–33.4)	32.0 (30.6–33.4)	32.0 (30.6–33.4)	32.0 (30.6–33.4)	32.0 (30.6–33.4)
	**Male**	**Both**	31.4 (30.3–32.6)	34.3 (33.1–35.5)	34.3 (33.1–35.5)	34.3 (33.1–35.5)	34.3 (33.1–35.5)	34.3 (33.1–35.5)
		**Rural**	24.6 (22.9–26.2)	26.8^††^ (25.1–28.5)	26.8^††^ (25.1–28.5)	26.8^††^ (25.1–28.5)	26.8^††^ (25.1–28.5)	26.8^††^ (25.1–28.5)
		**Urban**	34.4 (32.9–35.9)	37.6 (36.1–39.1)	37.6 (36.1–39.1)	37.6 (36.1–39.1)	37.6 (36.1–39.1)	37.6 (36.1–39.1)
**HDL-C domain**	**Both**	**Both**	69.3 (68.5–70.1)	71.1 (70.4–71.9)	71.1 (70.4–71.9)	71.1 (70.4–71.9)	71.1 (70.4–71.9)	71.1 (70.4–71.9)
		**Rural**	66.4 (65.1–67.6)	67.8 (66.6–69.1)	67.8 (66.6–69.1)	67.8 (66.6–69.1)	67.8 (66.6–69.1)	67.8 (66.6–69.1)
		**Urban**	70.5 (69.5–71.5)	72.5 (71.6–73.5)	72.5 (71.6–73.5)	72.5 (71.6–73.5)	72.5 (71.6–73.5)	72.5 (71.6–73.5)
	**Female**	**Both**	73.6 (72.6–74.7)	75.8 (74.8–76.8)	75.8 (74.8–76.8)	75.8 (74.8–76.8)	75.8 (74.8–76.8)	75.8 (74.8–76.8)
		**Rural**	73.5‡‡ (71.8–75.1)	75.2‡‡ (73.6–76.8)	75.2‡‡ (73.6–76.8)	75.2‡‡ (73.6–76.8)	75.2‡‡ (73.6–76.8)	75.2‡‡ (73.6–76.8)
		**Urban**	73.7‡‡ (72.4–75.1)	76.0‡‡ (74.7–77.4)	76.0‡‡ (74.7–77.4)	76.0‡‡ (74.7–77.4)	76.0‡‡ (74.7–77.4)	76.0‡‡ (74.7–77.4)
	**Male**	**Both**	64.1 (62.9–65.3)	65.7 (64.5–66.8)	65.7 (64.5–66.8)	65.7 (64.5–66.8)	65.7 (64.5–66.8)	65.7 (64.5–66.8)
		**Rural**	58.3 (56.4–60.2)	59.5 (57.6–61.4)	59.5 (57.6–61.4)	59.5 (57.6–61.4)	59.5 (57.6–61.4)	59.5 (57.6–61.4)
		**Urban**	66.6 (65.1–68.1)	68.3 (66.9–69.8)	68.3 (66.9–69.8)	68.3 (66.9–69.8)	68.3 (66.9–69.8)	68.3 (66.9–69.8)
**BP domain**	**Both**	**Both**	41.3 (40.6–42.0)	44.1 (43.3–44.8)	44.1 (43.3–44.8)	44.1 (43.3–44.8)	44.1 (43.3–44.8)	44.1 (43.3–44.8)
		**Rural**	42.1‡ (40.8–43.3)	44.7‡ (43.4–45.9)	44.7‡ (43.4–45.9)	44.7‡ (43.4–45.9)	44.7‡ (43.4–45.9)	44.7‡ (43.4–45.9)
		**Urban**	40.9‡ (40.0–41.8)	43.8‡ (42.9–44.7)	43.8‡ (42.9–44.7)	43.8‡ (42.9–44.7)	43.8‡ (42.9–44.7)	43.8‡ (42.9–44.7)
	**Female**	**Both**	40.9† (39.9–41.9)	44.1† (43.1–45.1)	44.1† (43.1–45.1)	44.1† (43.1–45.1)	44.1† (43.1–45.1)	44.1† (43.1–45.1)
		**Rural**	43.7 (42.0–45.4)	46.8 (45.1–48.5)	46.8 (45.1–48.5)	46.8 (45.1–48.5)	46.8 (45.1–48.5)	46.8 (45.1–48.5)
		**Urban**	39.4 (38.2–40.7)	42.7 (41.4–43.9)	42.7 (41.4–43.9)	42.7 (41.4–43.9)	42.7 (41.4–43.9)	42.7 (41.4–43.9)
	**Male**	**Both**	41.8† (40.7–42.8)	44.1† (43.0–45.2)	44.1† (43.0–45.2)	44.1† (43.0–45.2)	44.1† (43.0–45.2)	44.1† (43.0–45.2)
		**Rural**	40.3‡‡‡ (38.5–42.1)	42.3 (40.5–44.1)	42.3 (40.5–44.1)	42.3 (40.5–44.1)	42.3 (40.5–44.1)	42.3 (40.5–44.1)
		**Urban**	42.5‡‡‡ (41.2−43.9)	45.1 (43.7–46.4)	45.1 (43.7–46.4)	45.1 (43.7–46.4)	45.1 (43.7–46.4)	45.1 (43.7–46.4)

**ATP III:** National Cholesterol Education Program Adult Treatment Panel III; **IDF:** International Diabetes Federation; **AHA/NHLBI:** American Heart Association/National Heart, Lung, and Blood Institute; **JIS:** Joint Interim Statement; **TGs:** Triglycerides; **HDL-C:** High Density Lipoprotein Cholesterol; **BP:** Blood Pressure; **MetS:** Metabolic Syndrome; **95% CI:** 95% Confidence Interval.

-Data are presented as point estimates (%) and 95% CI.

-P ≤ 0.05 was considered as statistically significant using linear regression analysis for each domain and MetS definitions. Statistically non-significant differences among the different groups are noted as following:

†*P >* 0.05 for female versus male.

††*P >* 0.05 for female versus male among rural residents.

†††P > 0.05 for female versus male among urban residents.

‡*P* > 0.05 for rural versus urban areas.

‡‡*P* > 0.05 for rural versus urban areas among females.

‡‡‡*P* > 0.05 for rural versus urban areas among males.

The proportion of the highest and the lowest sub-national age-standardized prevalence rates of MetS estimated by JIS and RIDF were 48.2% and 19.2%, respectively. As for ATP III, IDF, AHA/NHLBI, JIS, RIDF and RJIS, the rates are as high as 87%, 43%, 66%, 40%, 79%, and 60%, correspondingly. In line with the national prevalence rate of MetS (data available at https://vizit.report/en/index.html), the prevalence rate in most provinces was highest in the 65–69 age group.

**Fig 2:**
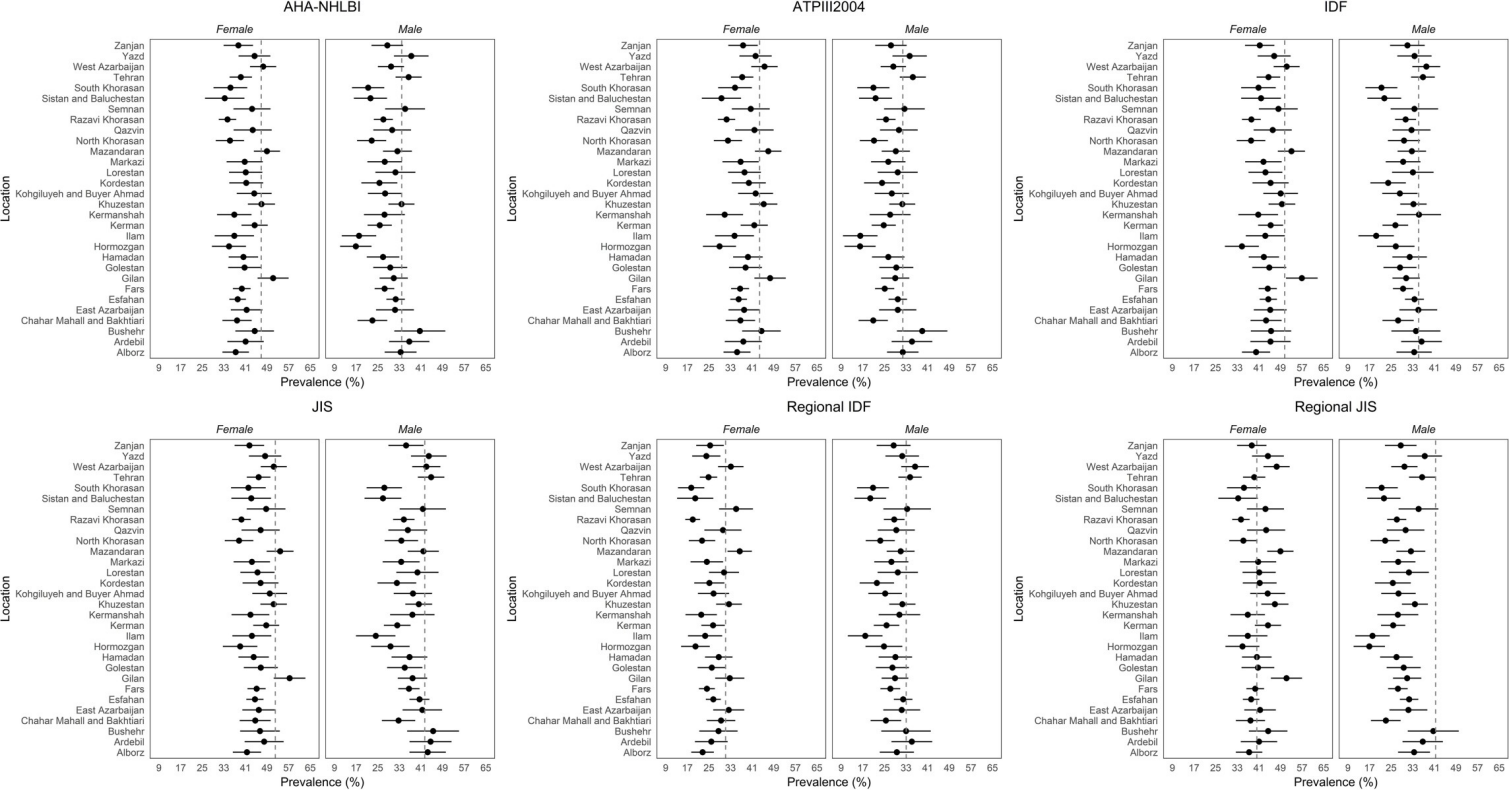
Gender and geographical distribution of sub-national prevalence of MetS based on six different definitions (age-standardized).

**Fig 3:**
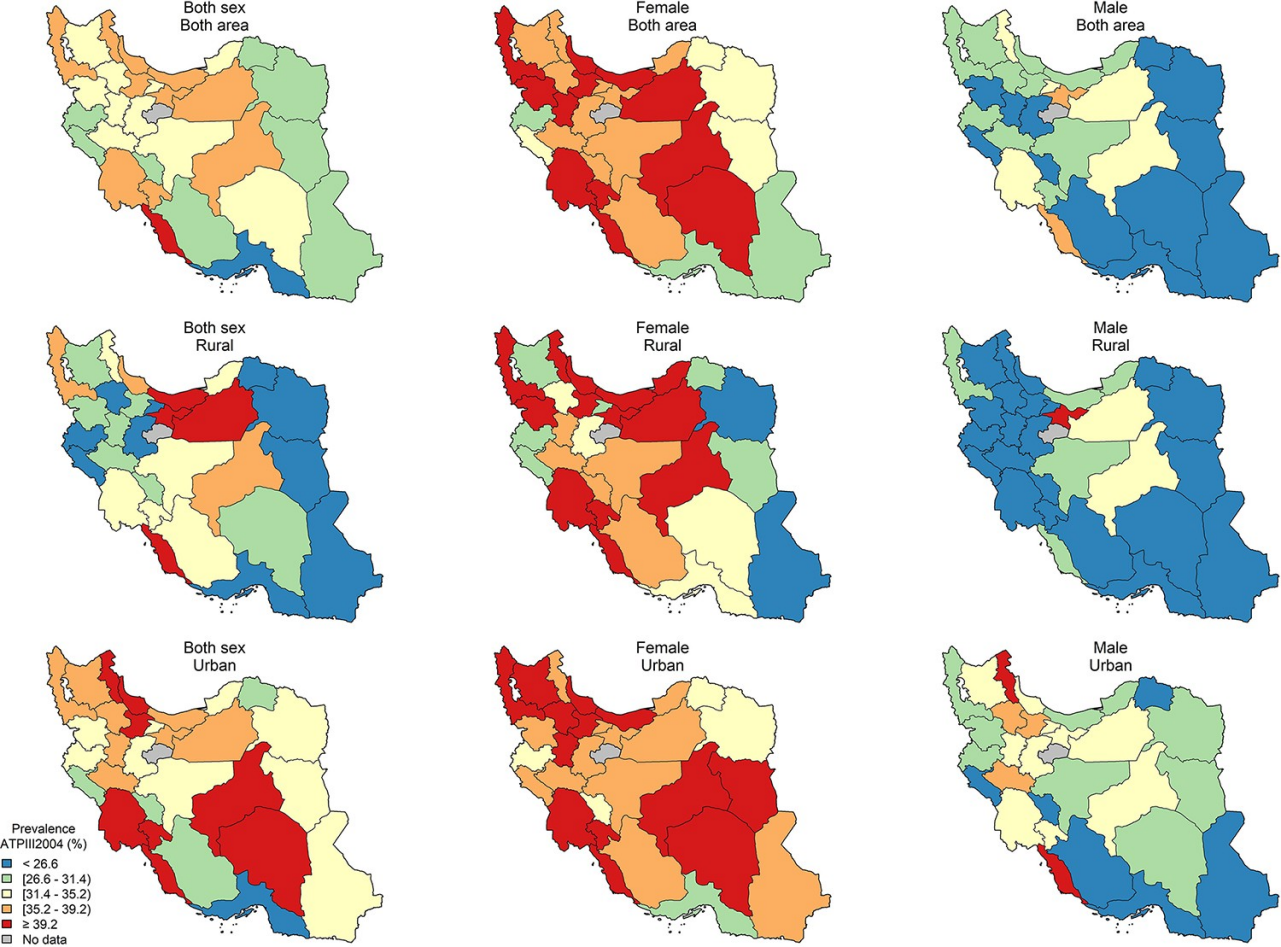
Geographical distribution of the prevalence of MetS by ATP III 2004.

**Fig 4:**
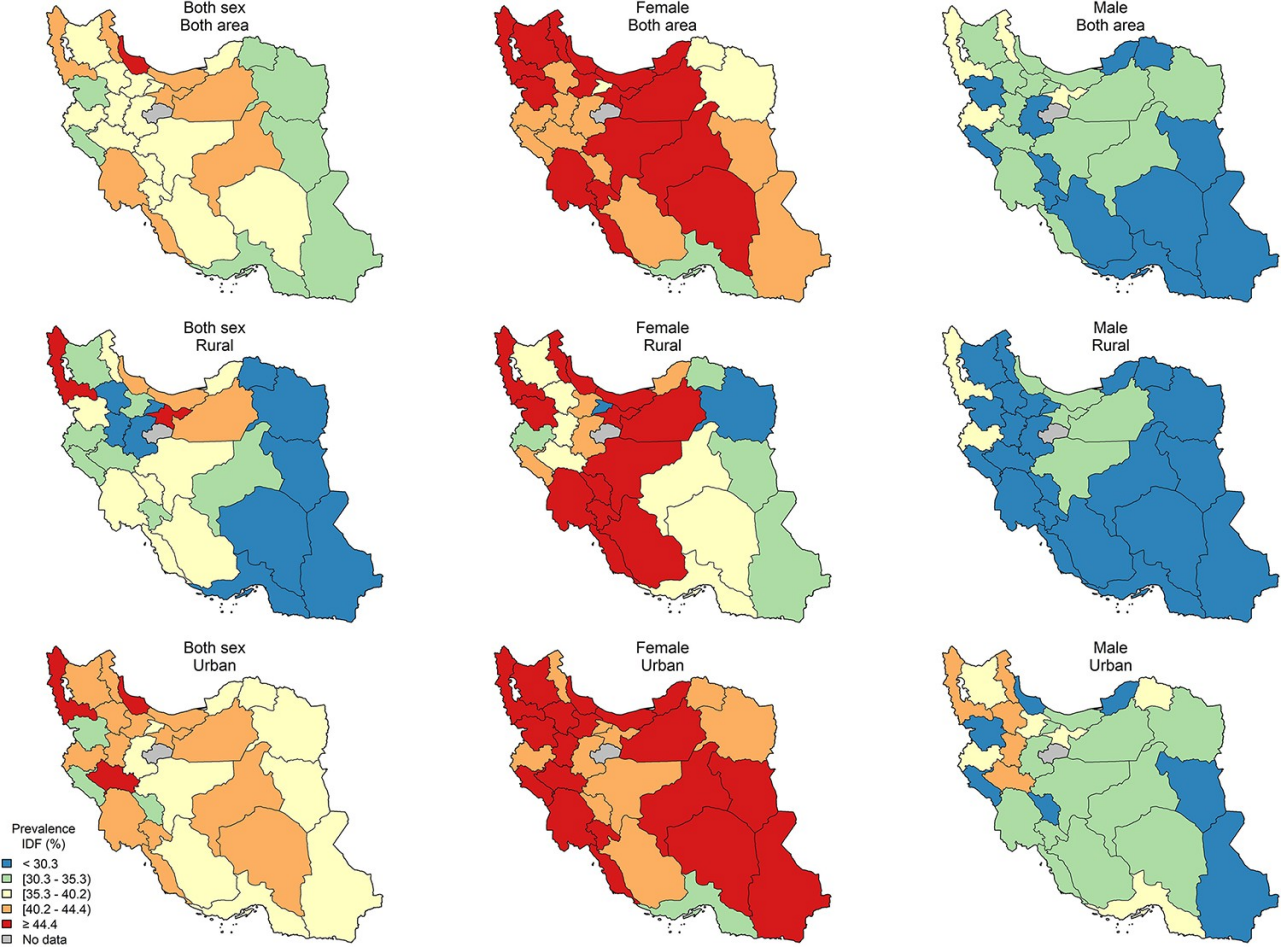
Geographical distribution of the prevalence of MetS by IDF.

**Fig 5:**
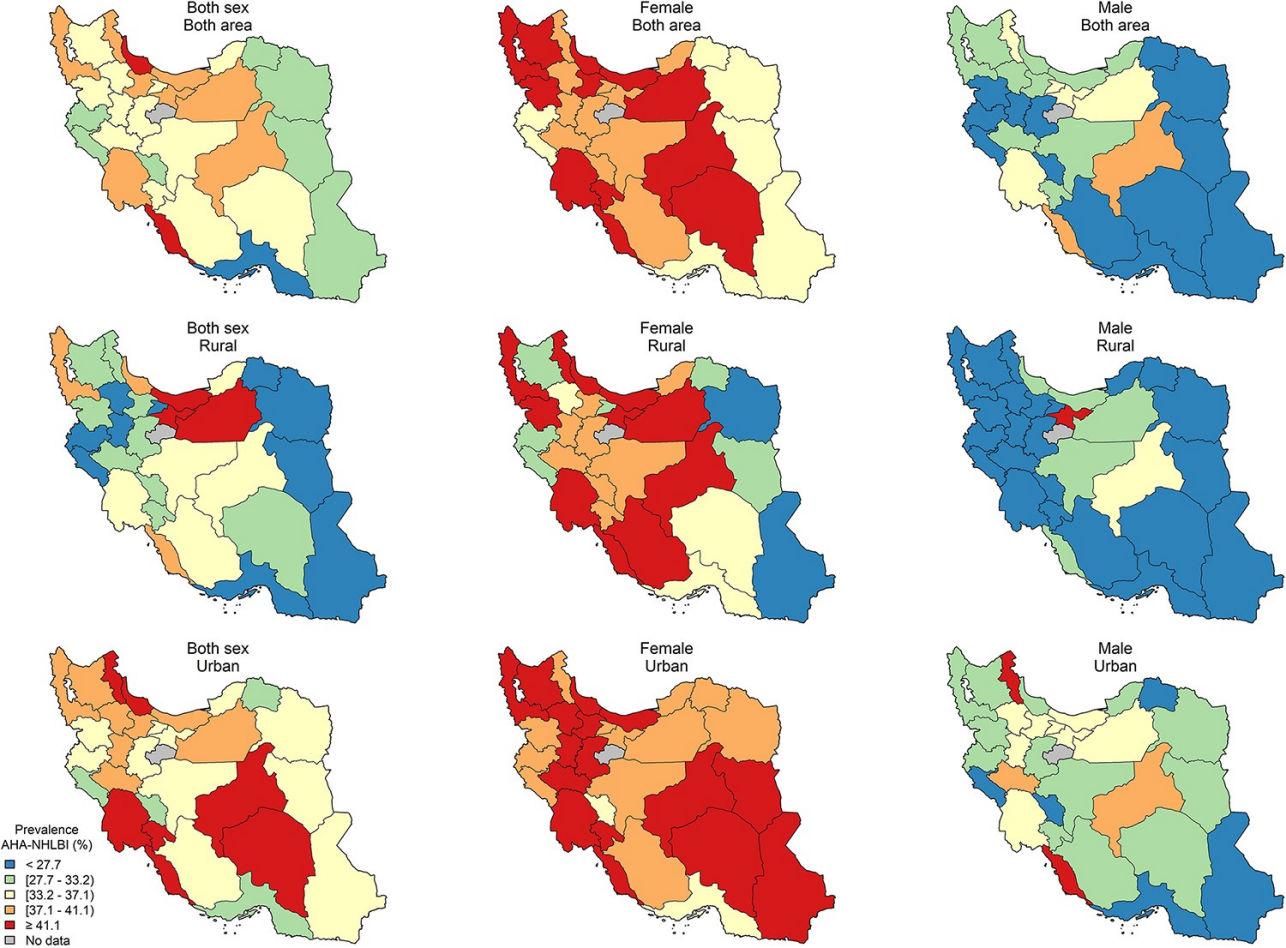
Geographical distribution of the prevalence of MetS by AHA/NHLBI.

**Fig 6:**
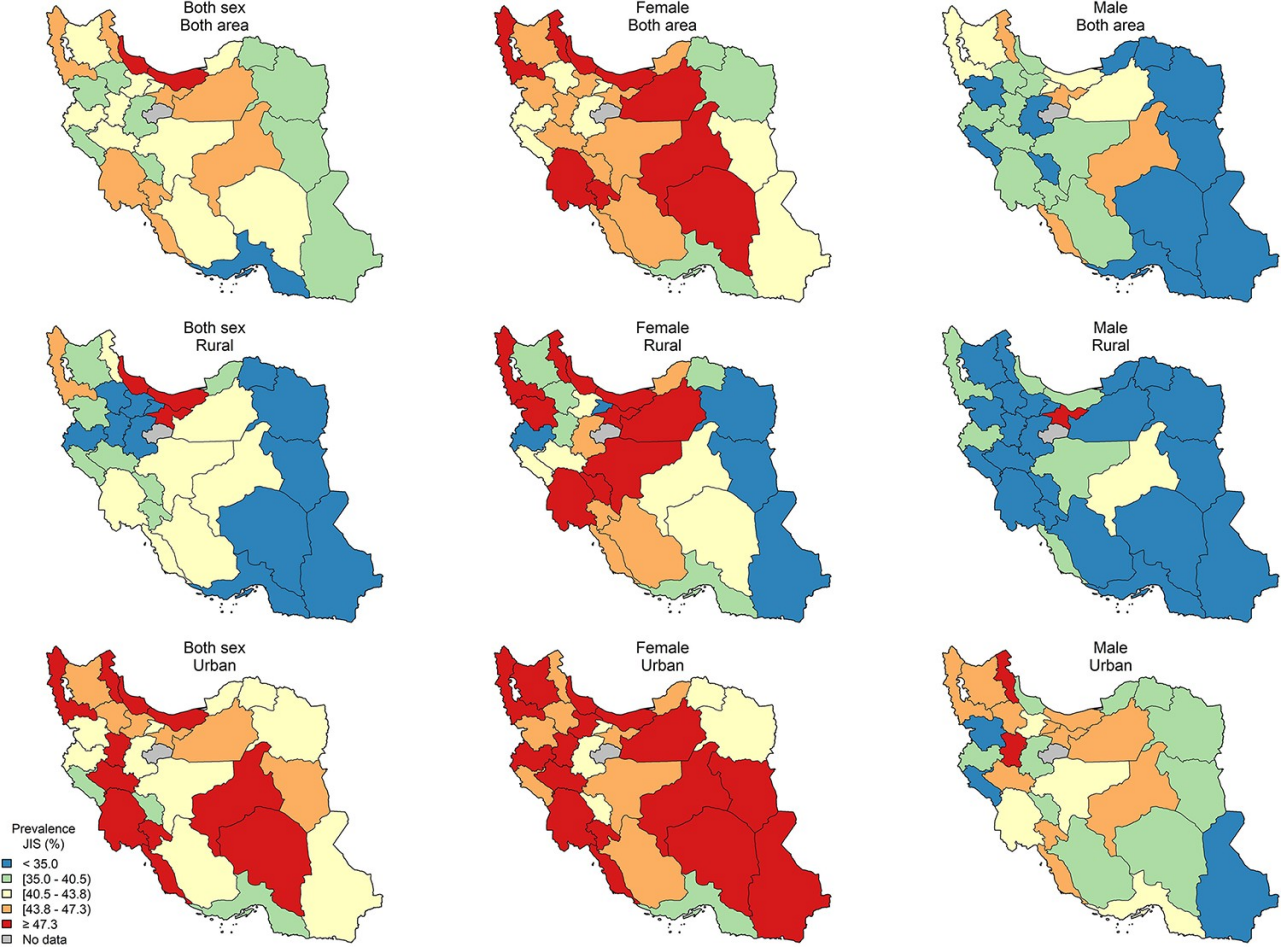
Geographical distribution of the prevalence of MetS by JIS.

**Fig 7:**
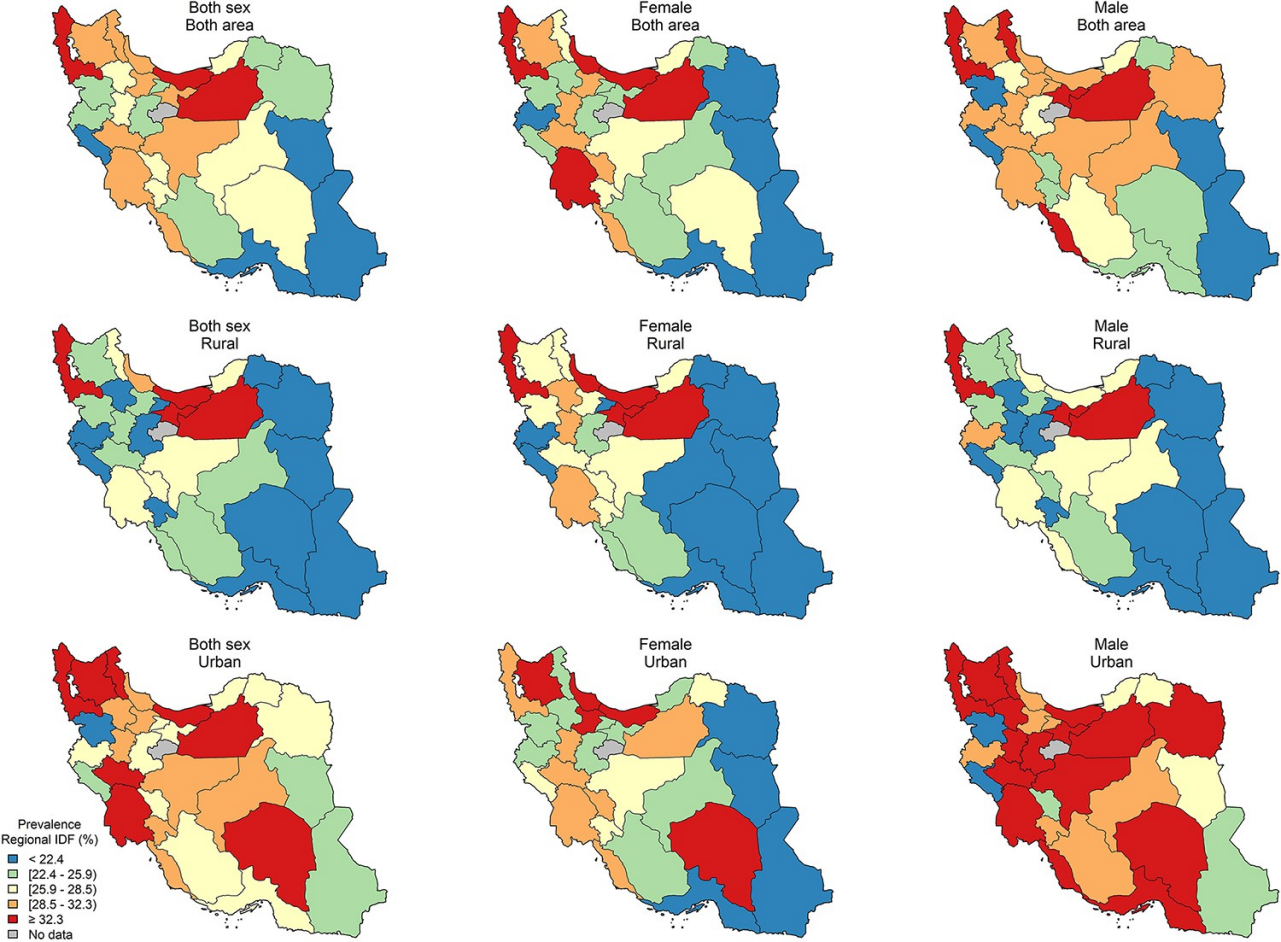
Geographical distribution of the prevalence of MetS by Regional IDF.

**Fig 8:**
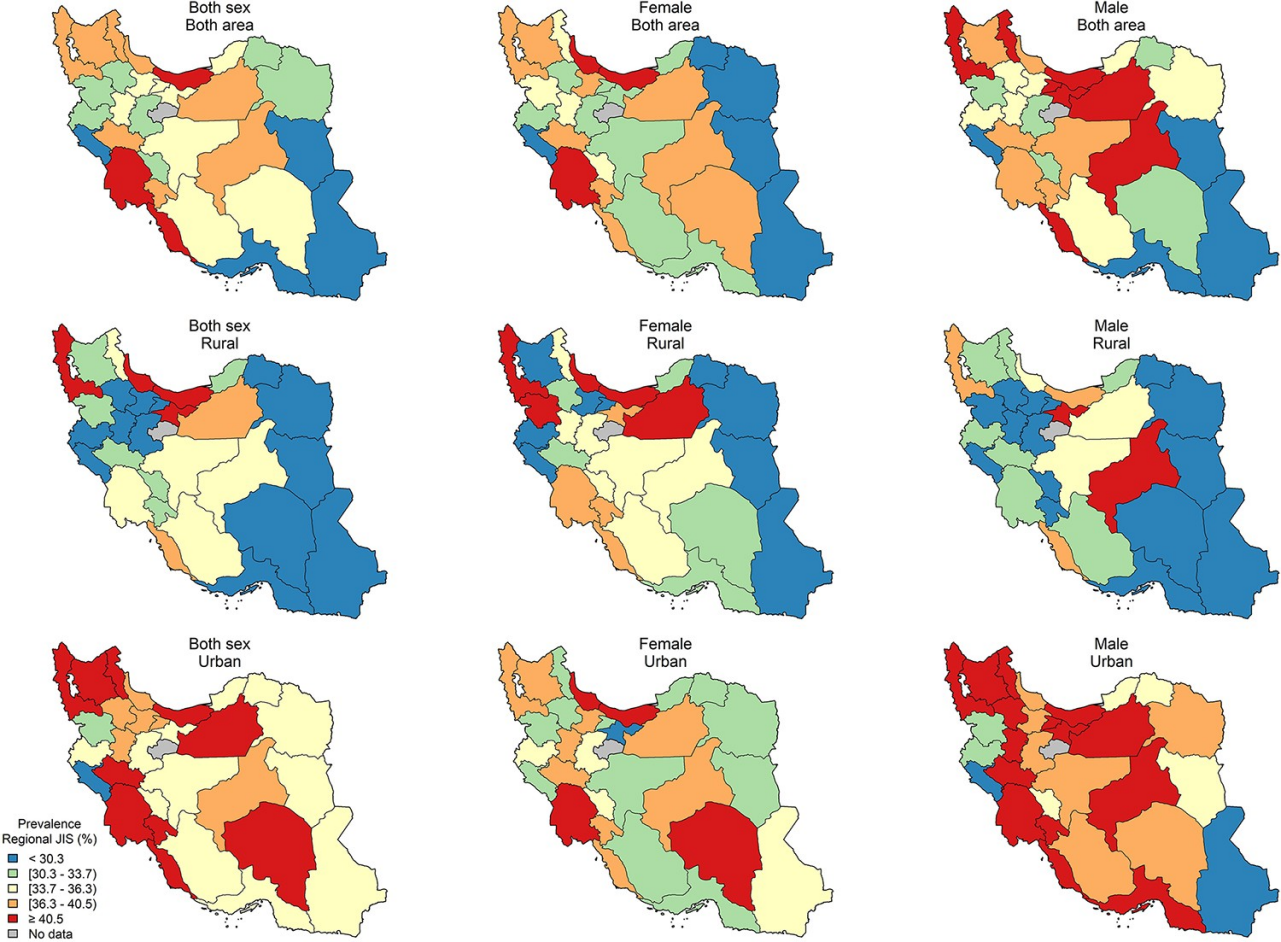
Geographical distribution of the prevalence of MetS by Regional JIS.

The age-standardized sub-national prevalence based on most of the used criteria (except for RIDF and RJIS) was higher among females in nearly all the provinces (Fig 2). According to the ATP III, IDF, AHA/NHLBI, and JIS definitions, there rates were higher in all the provinces among females and in urban populations (Figs 3–6). On the other hand, the rates based on RIDF and RJIS, presented in Figs 7 and 8, were shown to be higher among males and in urban residents.

The kappa agreement values between different definition criteria are presented in S1 Table. The lowest consistency (15.6%) was noted between the subjects determined to have MetS by the JIS criteria, but not the RIDF criteria (Table 6).

**Table 6:** Inconsistency (%) between different definitions of MetS.

Sex	MetS definition	*ATP III*^*-*^	*IDF*^*-*^	*AHA/NHLBI*^*-*^	*JIS*^*-*^	*Regional IDF*^*-*^	*Regional JIS*^*-*^
Both	***ATP III***^***+***^		3.6 (3.3–4.0)	0	0	11.2 (10.7–11.8)	3.3 (3.0–3.7)
	***IDF***^***+***^	8.9 (8.4–9.4)		6.7 (6.3–7.2)	0	11.5 (10.9–12.0)	6.8 (6.4–7.3)
	***AHA/NHLBI***^***+***^	2.6 (2.3–2.9)	4.1 (3.8–4.5)		0	12.3 (11.8–12.9)	3.6 (3.2–3.9)
	***JIS***^***+***^	9.4 (8.8–9.9)	4.1 (3.8–4.5)	6.7 (6.3–7.2)		15.6 (15.0–16.2)	6.8 (6.4–7.3)
	***Regional IDF***^***+***^	5.0 (4.6–5.4)	0	3.5 (3.2–3.8)	0		0
	***Regional JIS***^***+***^	5.8 (5.4–6.3)	4.1 (3.8–4.5)	3.5 (3.2–3.8)	0	8.8 (8.3–9.3)	
Female	***ATP III***^***+***^		1.1 (0.9–1.3)	0	0	14.2 (13.4–15.1)	6.1 (5.5–6.7)
	***IDF***^***+***^	7.9 (7.2–8.6)		5.2 (4.7–5.7)	0	19.4 (18.4–20.3)	11.8 (11.0–12.6)
	***AHA/NHLBI***^***+***^	3.1 (2.6–3.6)	1.5 (1.2–1.8)		0	15.7 (14.8–16.6)	6.6 (6.0–7.2)
	***JIS***^***+***^	8.3 (7.6–9.0)	1.5 (1.2–1.8)	5.2 (4.7–5.7)		20.9 (19.9–21.9)	11.8 (11.0–12.6)
	***Regional IDF***^***+***^	1.6 (1.3–1.9)	0	0	0		0
	***Regional JIS***^***+***^	2.6 (2.2–3.1)	1.5 (1.2–1.8)	0	0	9.1 (8.4–9.8)	
Male	***ATP III***^***+***^		6.6 (6.0–7.3)	0	0	7.7 (7.0–8.4)	0
	***IDF***^***+***^	10.0 (9.2–10.8)		8.6 (7.8–9.3)	0	2.1 (1.7–2.5)	1.0 (0.7–1.3)
	***AHA/NHLBI***^***+***^	2.1 (1.7–2.5)	7.2 (6.6–7.9)		0	8.4 (7.7–9.1)	0
	***JIS***^***+***^	10.6 (9.8–11.4)	7.2 (6.6–7.9)	8.6 (7.8–9.3)		9.4 (8.6–10.1)	1.0 (0.7–1.3)
	***Regional IDF***^***+***^	8.9 (8.2–9.7)	0	7.6 (6.9–8.2)	0		0
	***Regional JIS***^***+***^	9.6 (8.9–10.4)	7.2 (6.6–7.9)	7.6 (6.9–8.2)	0	8.4 (7.7–9.1)	

**ATP III:** National Cholesterol Education Program Adult Treatment Panel III; **IDF:** International Diabetes Federation; **AHA/NHLBI:** American Heart Association/National Heart, Lung, and Blood Institute; **JIS:** Joint Interim Statement.

-Data are presented as point estimates (%) and 95% CI.

Sex pattern of the highest inconsistency was shown between those with MetS diagnosed by JIS but not by RIDF (20.9%) or ATP III (10.6%), in females and males, respectively.

However, similar low inconsistency rate were observed between females diagnosed by ATP III, but not by IDF (1.1%), and males diagnosed by IDF or JIS, but not by RJIS (1.0%). Compared with RIDF, RJIS was reported to be capable of diagnosing more Iranians with MetS (9.1% in females, and 8.4% in males) (Table 6).

## Discussion

This nationwide study was conducted to estimate the prevalence rate of MetS in Iran. The results revealed that the national burden and the prevalence of MetS are alarming, particularly among females, in urban populations, and the 65–69 age group. The highest prevalence rate was observed using the JIS criteria. The two most common components of MetS reported in this population were increased WC and reduced HDL-C.

In our study, the prevalence rate of MetS was considerably higher than that reported in most Middle Eastern countries and even other parts of the world. The prevalence of MetS for all ages is reported to be about 21.3% in China, 27.8% in Spain, and 34.3% in the US [6,22,23]. The overall prevalence of MetS in the Middle Eastern countries is reported to range from 16.7% and 18.9% in Saudi Arabia [7] to 26.5% and 33.7% in Qatar, based on ATP III and IDF criteria respectively [24]. The prevalence of MetS in 2013 was 31.3% by ATP III, 35.0% by IDF, 35.8% by AHA/NHLBI, and 41.8% by JIS criteria in the biggest city of Iran (Tehran) [25]. Perhaps, one of the likely reasons for the observed variations between countries could be attributed to the cutoff points used to define central obesity. In addition, WC, which is considered as the core component in defining MetS using the IDF criteria, is influenced by ethnicity-specific values. However, the optimal WC cut off points recommended by the National Committee of Obesity (≥95 cm for Iranians regardless of gender) [19] are higher than that suggested by the Europid for females and lower for males. This may result in a higher prevalence rate of MetS among males.

The growing trend in the prevalence rate of MetS by age in our study is in line with the first nationwide survey conducted in the same population [26]. On the other hand, in the latter study the majority of the affected individuals belonged to the 55–64 age group, our current results are consistent with the findings of our neighboring country i.e. Turkey [27].

Due to the remarkable aging of the Iranian population in recent years and an increase in life expectancy, a rise in the prevalence of MetS among the elderly was expected. In addition, the increase in the number of post-menopausal women due to aging can also be associated with the accumulation of excess abdominal fat and the surging trend noted among females [28]. In proportion to the increase reported in MetS rates, the prevalence of other NCDs risk factors such as diabetes and obesity has also increased in not only our country but also the region [29,30].

The gender pattern of the national and sub-national MetS prevalence rates of all ages among our studied subjects was inconsistent with that of studies conducted in other Middle Eastern or non-Middle Eastern countries using similar definition criteria [8,31,32]. This gender disparity might be attributed to multiparity, low levels of HDL-C, and sedentary lifestyle among females, as opposed to males.

In corroboration with the findings of two systematic reviews on the prevalence of MetS in the Middle East countries and Asia-pacific region, a higher prevalence rate was noted in the urban population of this study [33,34]. Furthermore, an association between MetS prevalence and education level was observed. As opposed to lower education levels, higher education levels appeared to be correlated with lower engagement in harmful habits such as unhealthy eating habits, sedentary lifestyle, smoking, and low access to healthcare services [35]. Overall, socio-environmental factors such as rapid population growth, migration from rural to urban areas, and unhealthy lifestyles were recognized as some of the factors associated with the increased risk of MetS and its components [36].

The most common components of MetS in our study were similar to those from other countries in the region [37]. The reported prevalence of dyslipidemia in Iran was about 43.9% [38], which was higher than the values reported in Turkey (31.8%) and lower than those reported in the Arab populations e.g. Jordan (62.7%) [37]. These reports were comparable to the ones reported from non-Middle Eastern countries such as the US (35.0% of males and 40.4% of females) in a survey of 5455 adults conducted in the 2013–2014 NHANES survey [39].

The reason behind these variations might be ascribed to an ethnic predisposition to insulin resistance coupled with the high-caloric unhealthy diets in Iran similar to other Middle Eastern countries [40,41]. However, the similarity in gender differences could be partly related to the sedentary lifestyle that is more prevalent among females than in males. Creer et al. conducted a longitudinal study and found significant effects of physical inactivity, sedentary behavior, and cardiorespiratory fitness (CRF) on the development of MetS [42]. A rise in the rate of physical inactivity, from 15.5% in 2007 to 21.5% in 2011, was reported in a study from Iran [43]. Another reason of these findings may be attributed to rapid aging of the Iranian population that has resulted in a nearly four times increase in their mean age over the past 60 years [44].

Like many studies, our study has its strengths and limitations. The main strength of our survey was the assessment of the prevalence rate of MetS in a large population-based sample of both sexes and across a broad age spectrum, in urban and rural areas of all provinces of Iran. Furthermore, our results present the sub-national age-standardized prevalence rate, which has been reported by a few studies so far. Moreover, it provides the possibility to compare different provinces in terms of sex and age patterns of MetS prevalence. Therefore, policymakers can utilize the results to integrate appropriate preventive programs. The third strength is the comparison of prevalence rates by the use of various definition criteria and estimation of prevalence rates of MetS’ components based on data analysis in a unique lab center (NCDRC) which improves the accuracy of the results.

The main limitation of this study is its cross-sectional nature. Future studies are required to offer different insights into MetS.

## Conclusion

In conclusion, our findings indicate an alarming prevalence of MetS, both at national and sub-national levels, particularly among females, in the urban population, and the elderly. The highest prevalence rate observed using the JIS criteria. This could be indicated to this fact that the JIS criteria is more appropriate for our country due to its high coverage of population at risk of NCDs. The management and prevention of MetS requires multiple actions through several modalities, including raising public awareness on NCDs risk factors, lifestyle modifications, and pharmacological interventions to modify risk factors such as lipid abnormalities. Furthermore, a unique universal criterion with ethnicity-specific values for waist circumference is needed for the identification of MetS and more accurate comparison of its prevalence rates in different countries.

## Supporting information

S1 Table Kappa values between different definitions of MetS. (DOCX)

S1 File (PDF)
