# Supplementary material for: A nationwide study of metabolic syndrome prevalence in Iran; a comparative analysis of six definitions
Source: PLoS One. 2021 Mar 3;16(3):e0241926. doi: 10.1371/journal.pone.0241926 (PMC7928520; doi:10.1371/journal.pone.0241926)
Supplement: S1 Table — (DOCX) [file pone.0241926.s001.docx]

**Table S1: Kappa values between different definitions of MetS**

| **Sex** |  | *ATP III* | *IDF* | *AHA/NHLBI* | *JIS* | *Regional IDF* | *Regional JIS* |
| --- | --- | --- | --- | --- | --- | --- | --- |
| Both | *ATP III* |  | 0.74 (0.006) | 0.95 (0.003) | 0.81 (0.005) | 0.65 (0.007) | 0.81 (0.006) |
|  | *IDF* | 0.74 (0.006) |  | 0.78 (0.006) | 0.92 (0.004) | 0.76 (0.006) | 0.78 (0.006) |
|  | *AHA/NHLBI* | 0.95 (0.003) | 0.78 (0.006) |  | 0.86 (0.005) | 0.66 (0.007) | 0.85 (0.005) |
|  | *JIS* | 0.81 (0.005) | 0.92 (0.004) | 0.86 (0.005) |  | 0.68 (0.006) | 0.86 (0.005) |
|  | *Regional IDF* | 0.65 (0.007) | 0.76 (0.006) | 0.66 (0.007) | 0.68 (0.006) |  | 0.81 (0.005) |
|  | *Regional JIS* | 0.81 (0.006) | 0.78 (0.006) | 0.85 (0.005) | 0.86 (0.005) | 0.81 (0.005) |  |
| Female | *ATP III* |  | 0.82 (0.007) | 0.94 (0.005) | 0.84 (0.007) | 0.67 (0.009) | 0.82 (0.008) |
|  | *IDF* | 0.82 (0.007) |  | 0.87 (0.006) | 0.97 (0.003) | 0.61 (0.009) | 0.74 (0.008) |
|  | *AHA/NHLBI* | 0.94 (0.005) | 0.87 (0.006) |  | 0.90 (0.005) | 0.68 (0.009) | 0.87 (0.006) |
|  | *JIS* | 0.84 (0.007) | 0.97 (0.003) | 0.90 (0.005) |  | 0.59 (0.009) | 0.77 (0.008) |
|  | *Regional IDF* | 0.67 (0.009) | 0.61 (0.009) | 0.68 (0.009) | 0.59 (0.009) |  | 0.80 (0.007) |
|  | *Regional JIS* | 0.82 (0.008) | 0.74 (0.008) | 0.87 (0.006) | 0.77 (0.008) | 0.80 (0.007) |  |
| Male | *ATP III* |  | 0.63 (0.011) | 0.95 (0.005) | 0.78 (0.008) | 0.62 (0.011) | 0.79 (0.008) |
|  | *IDF* | 0.63 (0.011) |  | 0.65 (0.010) | 0.85 (0.007) | 0.95 (0.005) | 0.83 (0.008) |
|  | *AHA/NHLBI* | 0.95 (0.005) | 0.65 (0.010) |  | 0.82 (0.008) | 0.64 (0.011) | 0.84 (0.007) |
|  | *JIS* | 0.78 (0.008) | 0.85 (0.007) | 0.82 (0.008) |  | 0.80 (0.008) | 0.98 (0.003) |
|  | *Regional IDF* | 0.62 (0.011) | 0.95 (0.005) | 0.64 (0.011) | 0.80 (0.008) |  | 0.82 (0.008) |
|  | *Regional JIS* | 0.79 (0.008) | 0.83 (0.008) | 0.84 (0.007) | 0.98 (0.003) | 0.82 (0.008) |  |

**Legend: ATP III:** National Cholesterol Education Program Adult Treatment Panel III; **IDF:** International Diabetes Federation; **AHA/NHLBI:** American Heart Association/National Heart, Lung, and Blood Institute; **JIS:** Joint Interim Statement of the International Diabetes Federation Task Force on Epidemiology and Prevention

-Data are presented as point estimates with standard errors (SE).
